# Substantially improving the enantioconvergence of *Pv*EH1, a *Phaseolus vulgaris* epoxide hydrolase, towards *m*-chlorostyrene oxide by laboratory evolution

**DOI:** 10.1186/s12934-019-1252-4

**Published:** 2019-11-18

**Authors:** Xun-Cheng Zong, Chuang Li, Yao-Hui Xu, Die Hu, Bo-Chun Hu, Jia Zang, Min-Chen Wu

**Affiliations:** 1 Key Laboratory of Carbohydrate Chemistry and Biotechnology, Ministry of Education, School of Biotechnology, Jiangnan University, Wuxi, 214122 China; 2 The Affiliated Wuxi Matemity and Child Health Care Hospital of Nanjing Medical University, Wuxi, 214002 China; 3 Wuxi School of Medicine, Jiangnan University, Wuxi, 214122 China

**Keywords:** Epoxide hydrolase, Enantioconvergence, *m*-Chlorostyrene oxide, *m*-Chlorophenyl-1,2-ethanediol, Laboratory evolution, Substrate-binding pocket

## Abstract

**Background:**

Epoxide hydrolase can regioselectively catalyze the oxirane ring-opening hydrolysis of *rac*-epoxides producing the corresponding chiral diols. In our laboratory, a gene named *pveh1* encoding an EH from *Phaseolus vulgaris* was cloned. Although the directed modification of *Pv*EH1 was carried out, the mutant *Pv*EH1^Y3^ showed a limited degree of enantioconvergence towards racemic (*rac*-) *m*-chlorostyrene oxide (*m*CSO).

**Results:**

*Pv*EH1 and *Pv*EH1^Y3^ were combinatively subjected to laboratory evolution to further enhance the enantioconvergence of *Pv*EH1^Y3^ towards *rac*-*m*CSO. Firstly, the substrate-binding pocket of *Pv*EH1 was identified using a CAVER 3.0 software, and divided into three zones. After all residues in zones 1 and 3 were subjected to leucine scanning, two *E. coli* transformants, *E. coli*/*pveh1*^Y149L^ and /*pveh1*^P184L^, were selected, by which *rac*-*m*CSO was transformed into (*R*)-*m*-chlorophenyl-1,2-ethanediol (*m*CPED) having 55.1% and 27.2% *ee*_p_. Secondly, two saturation mutagenesis libraries, *E. coli*/*pveh1*^Y149X^ and /*pveh1*^P184X^ (X: any one of 20 residues) were created at sites Y149 and P184 of *Pv*EH1. Among all transformants, both *E. coli*/*pveh1*^Y149L^ (65.8% α_*S*_ and 55.1% *ee*_p_) and /*pveh1*^P184W^ (66.6% α_*S*_ and 59.8% *ee*_p_) possessed the highest enantioconvergences. Finally, the combinatorial mutagenesis was conducted by replacements of both Y149L and P184W in *Pv*EH1^Y3^, constructing *E. coli*/*pveh1*^Y3Z2^, whose α_*S*_ reached 97.5%, higher than that (75.3%) of *E. coli*/*pveh1*^Y3^. In addition, the enantioconvergent hydrolysis of 20 mM *rac*-*m*CSO was performed by *E. coli*/*pveh1*^Y3Z2^, giving (*R*)-*m*CPED with 95.2% *ee*_p_ and 97.2% yield.

**Conclusions:**

In summary, the enantioconvergence of *Pv*EH1^Y3Z2^ was successfully improved by laboratory evolution, which was based on the study of substrate-binding pocket by leucine scanning. Our present work introduced an effective strategy for the directed modification of enantioconvergence of *Pv*EH1.

## Background

Optically pure epoxides and vicinal diols, the highly value-added and versatile building blocks, were widely applied in pharmaceutical, fine chemical and agrochemical industries [1–3]. For example, (*R*)-*p*CPED was used for the synthesis of (*R*)-Eliprodil, a neuroprotective agent for the treatment of ischemic stroke, while (*R*)-*m*CPED for β3-adrenergic receptor agonists, such as SR 58611 and AJ-9677 [4]. With the ever-increasing environmental consciousness, the biocatalysis mediated by whole resting cells or enzymes was considered to be an alternative to chemocatalysis that required expensive chiral ligands and hazardous metals, such as Jacobsen’s asymmetric ring-opening hydrolysis and epoxidation [5, 6].

Epoxide hydrolases (EHs; 3.3.2.-), existing widely in nature, can enantioselectively and/or regioselectively catalyze the oxirane ring-opening hydrolysis of *rac*-epoxides, retaining epoxide enantiomers and/or producing the corresponding chiral diols. Based on the catalytic mechanisms of given EH-substrate pairs, the asymmetric hydrolysis of *rac*-epoxides was divided into two pathways: kinetic resolution and enantioconvergent hydrolysis [7]. The former can retain single epoxide enantiomers with an intrinsic limitation of 50% maximum yield, while the latter can produce optically pure vicinal diols with 100% theoretically yield [8].

The mono-enzymatic catalysis was an ideal bioprocess for preparing chiral diols via convergent hydrolysis of epoxides, but few naturally existing EHs had high and opposite regioselectivities towards (*R*)- and (*S*)-epoxides [9]. To completely and quickly hydrolyze *rac*-epoxide, EH applied in enantioconvergent hydrolysis also have to possess a low enantioselectivity (i.e., enantiomeric ratio, *E*). In view of the merits of mono-enzymatic catalysis and the shortage of highly enantioconvergent EHs, it is necessary to excavate novel EHs or to modify specific local configurations of the existing EHs by protein engineering [10]. Among the different gene mutagenesis techniques, saturation mutagenesis (SM) at sites lining the enzyme’s binding pocket has emerged as a particularly viable approach to improve selectivity [11, 12]. For example, through five rounds of iterative saturation mutagenesis of nine residues at sites lining in the substrate-binding pocket (SBP) of *Aspergillus niger* M200 EH (*An*EH), its best mutant, named H:12-A1, was selected, whose regioselectivity coefficients (α_*S*_ values) towards (*S*)-SO and -*p*CSO were higher than those of *An*EH, and by which *rac*-SO and -*p*CSO were transformed into (*R*)-PED and -*p*CPED, respectively, with over 70% enantiomeric excess (*ee*_p_) [13]. Several other research groups also reported the laboratory evolution of residues located in the SBP of EHs [14, 15].

Previously, to improve the enantioconvergence of *Pv*EH1 towards styrene epoxides, its laboratory evolution was carried out based on the computer-aided design. Among all tested mutants of *Pv*EH1, *Pv*EH1^L105I/M160A/M175I^ (renamed *Pv*EH1^Y3^), was selected (Additional file 1: Table S1). Its enantioconvergence towards *rac*-*m*CSO increased to 69.7% from 1.0% *ee*_p_ of *Pv*EH1 [10]. In our present work, the SBP of *Pv*EH1, identified and analyzed using a CAVER 3.0 software (http://www.caver.cz/), was divided into three zones. To substantially improve the enantioconvergence of *Pv*EH1, the zones 1 and 3 were subjected, in which all residues were subjected to leucine scanning [16], namely, substituting target residues with Leu, to identify the sites where Leu mutants had best EH enantioconvergence. Two saturation mutagenesis libraries, *E. coli*/*pveh1*^Y149X^ and /*pveh1*^P184X^, were constructed and screened to select the best substituted residues at their respective sites. Then, the combinatorial mutagenesis of *Pv*EH1^Y3^ was conducted by replacing its Y149 and P184 with the selected best substituted residues, thereby creating one five-site mutant of *Pv*EH1, named *Pv*EH1^Y3/Y149L/P184W^ or *Pv*EH1^Y3Z2^. Lastly, the enantioconvergent hydrolysis of *rac*-*m*CSO was carried out using resting cells of *E. coli*/*pveh1*^Y3Z2^.

## Materials and methods

### Strains, plasmids and chemicals

*E. coli* BL21(DE3) and pET-28a(+) (Novagen, Madison, WI) were used for the construction of recombinant plasmid and expression of *pveh1* or its variant, while PrimeSTAR HS DNA polymerase and DpnI endonuclease (TaKaRa, Dalian, China) were used for the leucine scanning and site-saturation mutagenesis of *pveh1* as well as the combinatorial mutagenesis of *pveh1*^Y3^. pET-28a-*pveh1* and -*pveh1*^Y3^ and their corresponding *E. coli/pveh1* and /*pveh1*^Y3^ were constructed and stored in our lab. *Rac*-*m*CSO, (*R*)- and (*S*)-*m*CPED were purchased from Energy (Shanghai, China). All other chemicals were of analytical purity.

### Homology modeling of *Pv*EH1 and identification of its SBP

**Fig. 1:**
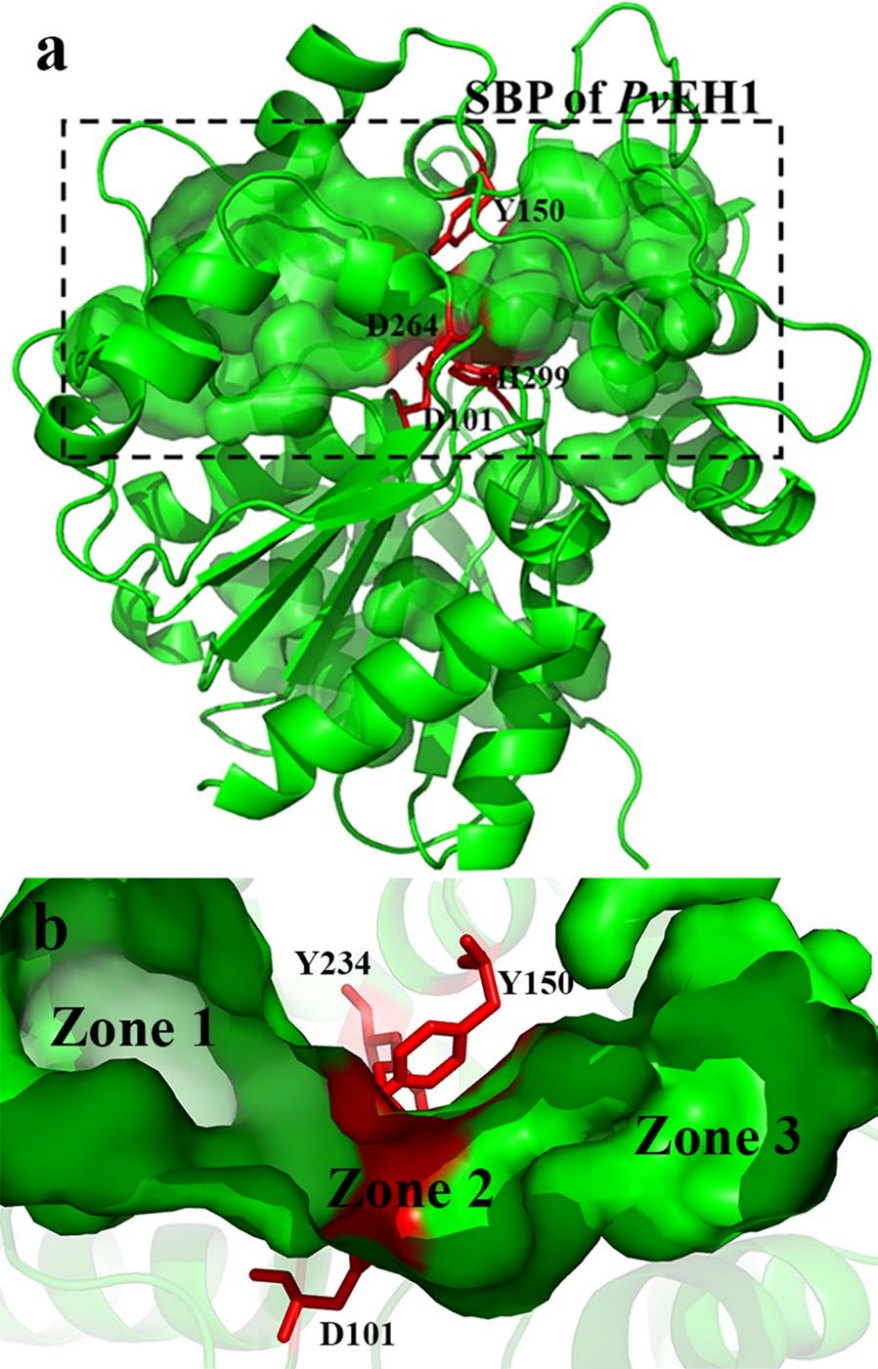
The SBP of *Pv*EH1 (**a**) and its three zones (**b**)

Using the known crystal structure of a *Vigna radiata* EH at 2.0 Å resolution (*Vr*EH1, PDB: 5XMD), sharing 87.4% identity with *Pv*EH1, as the template, the three-dimensional (3-D) structure of *Pv*EH1 was modeled using the MODELLER 9.11 program (http://salilab.org/modeller/) (Additional file 1: Figure S2), and subjected to molecular mechanics optimization by CHARMM27 force field in the GROMACS 4.5 package (http://www.gromacs.org/) (Additional file 1: Figure S3). The 3-D structure with the best geometry quality, which was validated by Structure Assessment in SWISS-MODEL (https://swissmodel.expasy.org/assess), was obtained, and visualized by a PyMOL software (http://pymol.org/) (Additional file 1: Figures S4 and S5). The SBP of a modeled *Pv*EH1’s 3-D structure was identified and analyzed using a CAVER 3.0 software, and artificially divided into three zones 1–3 (Fig. 1).

### Leucine scanning and site-saturation mutagenesis of *Pv*EH1

The zones 1 and 3 of *Pv*EH1’s SBP were subjected, in which all residues were subjected to leucine scanning. The variants of *pveh1* were designed by substituting the target residues-encoding codons with Leu-encoding codon, synthesized by Genewiz (Suzhou, China), and transformed into *E. coli* BL21, respectively, thereby constructing the corresponding *E. coli* transformants, such as *E. coli*/*pveh1*^Y149L^ and /*pveh1*^P184L^. Through screening, the specific residue sites where Leu mutants catalyzed the enantioconvergent hydrolysis of *rac*-*m*CSO with the highest *ee*_p_ values of (*R*)-*m*CPED were identified for the further studies.

Based on the results of leucine scanning, the saturation mutagenesis of a Y149- or P184-encoding codon in *pveh1* was carried out using a one-step whole-plasmid PCR method [17]. The primers of saturation mutagenesis were designed as reported previously [18], and synthesized by Sangon (Shanghai, China) as listed in Additional file 1: Table S2. Using pET-28a-*pveh1* as a template, the mutagenesis PCR was performed by PrimeSTAR HS DNA polymerase using a pair of primers, Y149X-F/Y149X-R or P184X-F/P184X-R, as following conditions: a denaturation at 95 °C for 4 min, 30 cycles of at 98 °C for 10 s, 55 °C for 15 s and 72 °C for 6 min, and an extra elongation at 72 °C for 10 min. The target PCR products, pET-28a-*pveh1*^Y149X^ or -*pveh1*^P184X^ (X: any one of 20 residues), were digested by DpnI at 37 °C for 6 h to decompose the methylated template DNA, and transformed into *E. coli* BL21(DE3), respectively, thereby constructing the mutagenesis library, *E. coli*/*pveh1*^Y149X^ or /*pveh1*^P184X^. Using both the *ee*_p_ of (*R*)-*m*CPED and *c* of *rac*-*m*CSO as indexes, *E. coli*/*pveh1*^Y149X^ and /*pveh1*^P184X^ were screened, respectively. The best *E. coli* transformant of each library at residue site Y149 or P184 of *Pv*EH1 was selected, expressing the EH mutant with the highest enantioconvergence towards *rac*-*m*CSO.

### Combinatorial site-directed mutagenesis of *Pv*EH1^Y3^

The combinatorial site-directed mutagenesis was designed by residue replacements of Y149L and P184W in *Pv*EH1^Y3^, and also carried out by one-step whole-plasmid PCR. The PCR primers were designed according to the nucleotide sequence of *pveh1*^Y3^ and codons encoding the selected mutation residues, and synthesized by Sangon (Additional file 1: Table S3). Using pET-28a-*pveh1*^Y3^ as the template, the first round of PCR was conducted using a pair of primers, Y149L-F/Y149L-R, under the same PCR conditions as described above. Thereafter, using the first-round PCR product as the template, the second round of PCR was carried out using another pair of primers, P184W-F/P184W-R. The target PCR product, pET-28a-*pveh1*^Y3/Y149L/P184W^ or -*pveh1*^Y3Z2^, was digested by DpnI, and transformed into *E. coli* BL21(DE3), thereby constructing one *E. coli* transformant, named *E. coli*/*pveh1*^Y3Z2^, harboring a five-site variant of *pveh1* or a two-site one of *pveh1*^Y3^.

### Analytic methods of HPLC and GC chromatographies

The activity of *Pv*EH1 or its mutant as well as the conversion ratio (*c*) of *rac*-*m*CSO defined as the ratio of its depleted concentration to initial one was assayed by high-performance liquid chromatography (HPLC), using an e2695 apparatus with an XBridge BEH C18 column (Waters, Milford, MA). The mobile phase of methanol/H_2_O (7:3, v/v) was used at 0.8 mL/min, and continuously monitored using a Waters 2489 UV–Vis detector at 220 nm. The generated diols (*R*)- and (*S*)-*m*CPED were analyzed by HPLC with a Chiralcel OD-H column (Daicel, Osaka, Japan). The *n*-hexane/isopropanol (9:1, v/v) was used as a mobile phase. Because (*R*)- and (*S*)-*m*CSO can not be separated by OD-H, they were assayed by chiral gas chromatography (GC), using a GC-2010 system (Shimadzu, Tokyo, Japan) with a CP-Chirasil-DEX CB column (Agilent, Santa Clara, CA) and a flame ionization detector. The injector and detector temperatures were 220 °C, while the column temperature was programmed from 110 to 190 °C at 10 °C/min. The nitrogen gas carrier was used at 3.0 mL/min.

### EH expression of *E. coli* transformant and EH activity assay

*E. coli* transformant harboring *pveh1* or its variant, such as *E. coli/pveh1* or /*pveh1*^Y3Z2^, was inoculated into LB medium supplemented with 100 μg/mL kanamycin, and cultured at 37 °C overnight as the seed culture. Then, the same fresh medium was inoculated with 2% (v/v) seed culture, and grown until OD_600_ reached 0.6–0.8. The expression of *Pv*EH1 or its mutant was induced by 0.5 mM IPTG at 20 °C for 10 h. The induced transformant cells were collected, and resuspended in 100 mM Na_2_HPO_4_–NaH_2_PO_4_ buffer (pH 7.0) to 100 mg wet cells/mL. *E. coli* BL21(DE3) transformed with pET-28a(+), named *E. coli*/pET-28a, was used as the negative control.

The hydrolytic conditions for the *Pv*EH1 or its mutant activity assay were as follows: 475 μL cell suspension of *E. coli* transformant, suitably diluted with 100 mM phosphate buffer (pH 7.0), was mixed with 25 μL 200 mM *rac*-*m*CSO, incubated at 25 °C for 10 min, and terminated by adding 2 mL methanol. The sample was assayed by HPLC with a C18 column. One EH activity unit (U) was defined as the amount of whole wet cells catalyzing the hydrolysis of 1 μmol *rac*-*m*CSO per minute under the given assay conditions.

### EH enantioconvergence assay

EH enantioconvergence assay was carried out as follows: 1.8 mL suitably diluted cell suspension was mixed with 200 μL 200 mM *rac*-*m*CSO and incubated at 25 °C. Aliquots of 100 μL sample were periodically drawn out, and extracted with 900 μL ethyl acetate for chiral HPLC analysis (or containing 1 mM *n*-hexanol as the internal standard for GC analysis). (*R*)- and (*S*)-*m*CPED were analyzed by chiral HPLC, while (*R*)- and (*S*)-CSO by GC. Both the *ee*_p_ of (*R*)-*m*CPED and *ee*_s_ of (*R*)-CSO were calculated with the equations: *ee*_p_ = [(*R*_p_ − *S*_p_)/(*R*_p_ + *S*_p_)] × 100% and *ee*_s_ = [(*R*_s_ − *S*_s_)/(*R*_s_ + *S*_s_)] × 100%, in which *R*_p_ and *S*_p_ were the concentrations of (*R*)- and (*S*)-product, while *R*_s_ and *S*_s_ were the concentrations of (*R*)- and (*S*)-substrate. The EH regioselectivity coefficients (β_*R*_ and α_*S*_) were applied to quantitatively evaluate the preference attacking on C_β_ (a less hindered terminal carbon in the oxirane ring) of (*R*)-epoxide and on C_α_ (a more hindered carbon) of (*S*)-epoxide, respectively [17]. Based on the above parameters, β_*R*_ and α_*S*_ were deduced by linear regression: *ee*_p_ = (α_*S*_ + β_*R*_ − 1) + [(β_*R*_ − α_*S*_) × *ee*_s_ × (1 − *c*)]/*c* [19].

### Enantioconvergent hydrolysis of *rac*-*m*CSO by *E. coli/pveh1*^Y3Z2^

As we using the whole cell as the biocatalyst, the regioselective hydrolysis of *rac*-*m*CSO, in a 5 mL 100 mM phosphate buffer (pH 7.0) system containing 20 mM *rac*-*m*CSO and 100 mg wet cells/mL of *E. coli*/*pv*eh1^Y3Z2^, was carried out at 25 °C until the *c* reached over 99%. During the hydrolytic course, aliquots of 100 μL reaction sample were drawn out, extracted with 900 μL ethyl acetate, and analyzed by chiral HPLC to calculate the *c* of *rac*-*m*CSO and *ee*_p_ of (*R*)-*m*CPED.

## Results and discussion

### 3-D structural analysis of *Pv*EH1 and its SBP identification

**Fig. 2:**
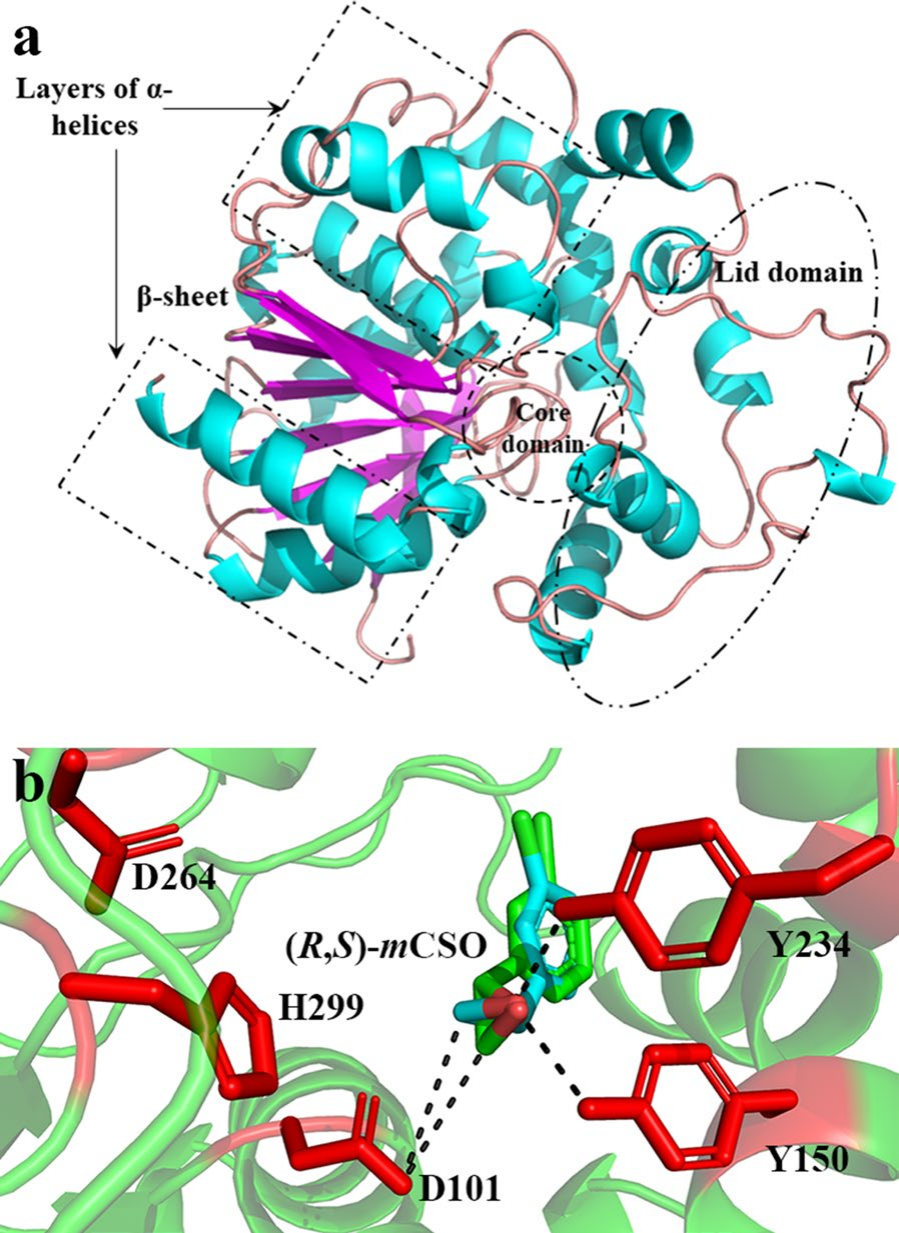
3-D structure of *Pv*EH1 (**a**) and its active center (**b**). The active center of was *Pv*EH1 made up by two polarizing tyrosine residues: Y150 and Y234 and catalytic triad D101-H299-D264

Most EHs belonged to α/β hydrolase superfamily, which consisted of a core domain, an α/β domain and a lid domain (Fig. 2a) [20]. Like 3-D structures of other EHs, that of *Pv*EH1 also contained a core domain, a β-sheet packed between two layers of 9 α-helices and a lid domain. Its active center was made up of two polarizing tyrosine residues: Y150 and Y234 and catalytic triad D101-H299-D264 (Fig. 2b). To avoid mutants losing catalytic activities, these five sites cannot be replaced by other residues. After being homologically modeled and optimized, 3-D structure of *Pv*EH1was analyzed to identify the SBP of *Pv*EH1. The SBP was located in the core domain and the lid domain mainly (Fig. 1a). It was reported that SBP can be divided according to the zone where the substrate binding with the enzyme [16]. Herein, as shown in Fig. 1b, the SBP of *Pv*EH1 was divided into three zones subjectively: zone 1 (residues A120-V126, M129, V135-G142 and Y149), zone 2 (residues D101, Y150, Y234, D264 and H299) and zone 3 (residues M160-T162, A171-M175 and R180-L186). Five residue sites in active center which displayed important roles in the catalytic reaction were in zone 2. Compared with zone 3, zone 1 involved in more residues (17 vs. 15). Similar with the SBP of LEH [15], that of *Pv*EH1 just looked like a dumbbell, which contained two big cavities and a tunnel connecting them.

### Leucine scanning of the SBP

**Fig. 3:**
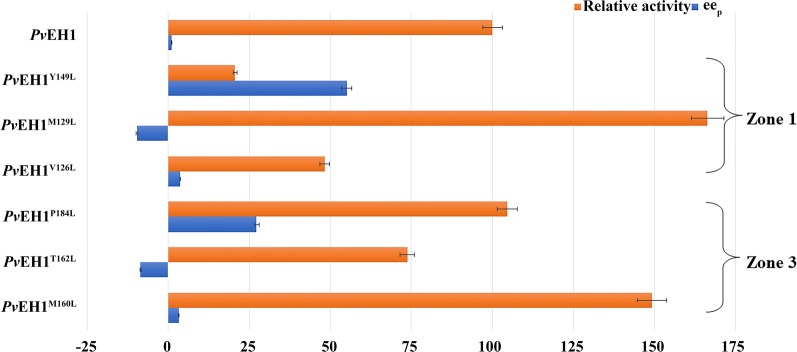
The enantioconvergence and relative activity part mutants from leucine scanning of zone 1 (contain *Pv*EH1^Y149L^, *Pv*EH1^M129L^ and *Pv*EH1^V126L^) and zone 3 (contain *Pv*EH1^P184L^, *Pv*EH1^T162L^ and *Pv*EH1^M160L^), where catalytic activity of *Pv*EH1 was used as a control

Amino acid substitutions at sites lining the SBP of EH may evolve its stereoselectivity [20]. As lacking the basic understanding of the prerequisites for regioselectivity in nucleophilic attack, each residue in zone 1 and zone 3 was replaced by leucine respectively to construct *E. coli* transformant expressing single-site mutant, except that the residue was leucine originally, for finding out the key sites influencing the enantioconvergence. The *ee*_p_ values and activities of all the mutants towards *rac*-*m*CSO were measured respectively. The results were outlined in Additional file 1: Table S4. Among all the mutants, *Pv*EH1^Y149L^ displayed a marked increase in enantioconvergence, and *ee*_p_ reached to 55.1%. On other side, residue replacement like M129L in zone 1 reversed the configuration of the main diol product partly (Fig. 3). The similar phenomenon was also observed in the reshaping of LEH SBP, in that case, a one-site mutant LEH^L114F^ favored the formation of (*R*,*R*)-diol, while LEH the formation of (*S*,*S*)-diol [15]. Compared with residue replacements in zone 1, those in zone 3 had smaller effects on the catalytic activities (Fig. 3). For example, different from mutant *Pv*EH1^Y149L^, *Pv*EH1^P184L^ had an increased enantioconvergence and its activity was not decreased. By the way, mutant *Pv*EH1^M129L^ displayed the highest catalytic activity, while mutant *Pv*EH1^Y149L^ the lowest except *Pv*EH1^P137L^ (no activity). There was an increase in the enantioconvergence of mutant *Pv*EH1^Y149L^, but under the comparison with other studies which have been reported, it is still not ideal enough [21, 22].

### Saturation mutagenesis of Y149 or P184 in *Pv*EH1

To figure out which residues at sites 149 and 184 are most beneficial for improving the enantioconvergence of *Pv*EH1, saturation mutagenesis libraries *E. coli*/*pveh1*^Y149X^ and *E. coli*/*pveh1*^P184X^ were constructed and screened. According to the “22c-trick” method reported by Kille et al. [18] a special mixture of three primers was employed to create a degeneracy of 22 unique codons coding for the 20 amino acids. To ensure a full coverage of potential mutants, well above a theoretical coverage of > 95%, all libraries were oversampled at least threefold. That is, 66 *E. coli* transformants from each library were needed. All transformants were screened by HPLC to select the highest mutant in *ee*_p_ value, then confirmed by DNA sequencing.

**Fig. 4:**
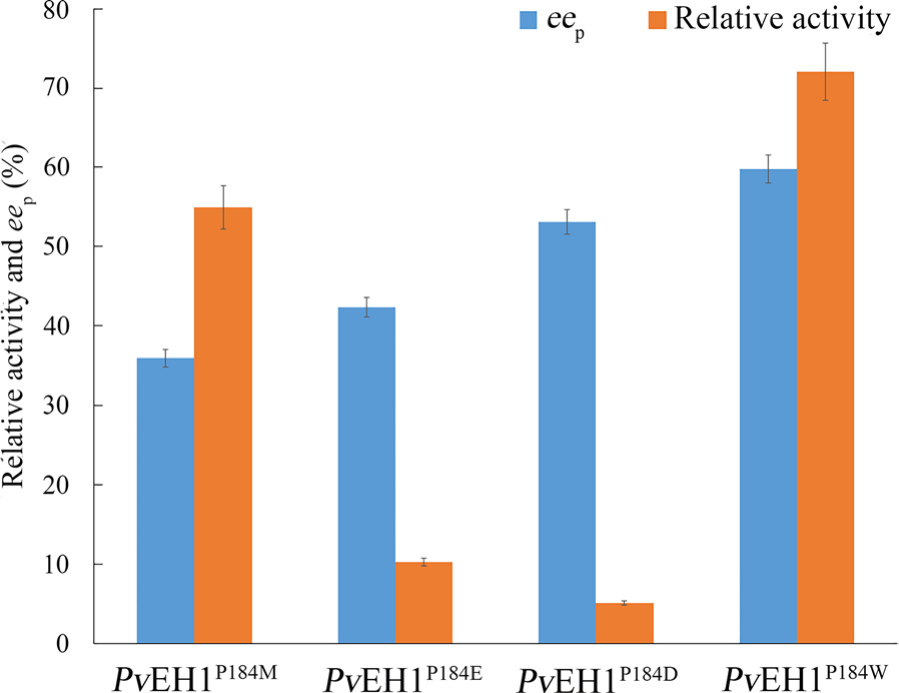
The screening of *Pv*EH1^P184X^ in for the *c* of *rac*-*m*CSO and *ee*_p_ of (*R*)-*m*CPED. After DNA sequencing, four best mutants (*Pv*EH1^P184M^, *Pv*EH1^P184E^, *Pv*EH1^P184D^ and *Pv*EH1^P184W^) were confirmed. The *ee*_p_ and *c* by these four were measured

As a result, in saturation mutagenesis library *E. coli*/*pveh1*^P184X^, four *E. coli* transformants with an over 35% in *ee*_p_ value were obtained, that is, *E. coli*/*pveh1*^P184E^, *E. coli*/*pveh1*^P184D^, *E. coli*/*pveh1*^P184M^ and *E. coli*/*pveh1*^P184W^, whose P184-encoding codon (CCT) was verified to be mutated to E-, D-, M- and W-encoding codons (GAG, GAT, ATG and TGG). As shown in Fig. 4, *E. coli*/*pveh1*^P184E^ and *E. coli*/*pveh1*^P184D^ could not transform 10 mM *rac*-*m*CSO completely, while *E. coli*/*pveh1*^P184M^ and *E. coli*/*pveh1*^P184W^ could. *E. coli*/*pveh1*^P184W^ displayed the highest enantioconvergence in saturation mutagenesis library *E. coli*/*pveh1*^P184X^. The *ee*_p_ of (*R*)-*m*CPED catalyzed by *E. coli*/*pveh1*^P184W^ was 59.8%, which was nearly 58-fold higher than that by *Pv*EH1. Unfortunately, most *E. coli* transformants in saturation mutagenesis library *E. coli*/*pveh1*^Y149X^ displayed no catalytic activity towards *m*CSO except *E. coli*/*pveh1*^Y149L^. Taking into consideration of studies on active areas in *Vr*EH2 and *St*EH1 [21, 23], it is likely that amino acid sites around site 149 (including 149) may display important roles in the catalytic activity. On the other hand, the exchanges (Y149L and P184W) were crucial for the regioselectivity regulation [13].

The regioselectivity coefficients (β_*R*_ and α_*S*_) were applied to quantitatively evaluate the preference attacking on C_β_ of (*R*)-epoxide which could afford the diol of unchanged (*R*)-configuration, and on C_α_ of (*S*)-epoxide which could lead to the (*R*)-diol by inversion of configuration. It was necessary to determine regioselectivity coefficients which were helpful to understand the mechanism of enantioconvergence [8]. The α_*S*_ values of mutants *Pv*EH1^Y149L^ and *Pv*EH1^P184W^ increased from 10.3% to 65.8% and 66.6%, which straight contributed to the high enantioconvergence. But they were still lower than that of *Pv*EH1^Y3^ (Table 1).

**Table 1:** Results of measurements of regioselectivity coefficients

EH	β_*R*_/%	α_*S*_/%	*ee*_p_/%
*Pv*EH1	94.1	10.3	1.0 ± 0.1
*Pv*EH1^Y149L^	89.2	65.8	55.1 ± 1.7
*Pv*EH1^P184W^	93.2	66.6	59.8 ± 1.7
*Pv*EH1^Y3^	94.4	75.3	69.7 ± 3.3
*Pv*EH1^Y3Z2^	97.9	97.5	96.1 ± 2.9

### Residue replacements of both Y149L and P184 W in *Pv*EH1^Y3^

According to the research of Ye et al. [10] mutant *Pv*EH1^Y3^ displayed a limited enantioconvergence towards *m*CSO (*ee*_p_ = 69.7%). But it still did not meet the requirement of production of (*R*)-*m*CPED. For further enhancing the enantioconvergence, we took the advantage of a cooperative mutational effect from replacements Y149L and P184W [24]. Mutant *Pv*EH1^Y3Z2^ (containing residues replacements: L105I, M160A, M175I, Y149L and P184W) was engineered, using *Pv*EH1^Y3^ as the template. The enantioconvergence of mutant *Pv*EH1^Y3Z2^ was 94-fold higher than that of *Pv*EH1 (*ee*_p_ 1.0%). Compared regioselectivity coefficients of *Pv*EH1^Y3Z2^ with those of *Pv*EH1, it was observed that almost all the improvement in enantioconvergence was contributed by the increase of α_*S*_ and there was no obviously change in the β_*R*_. The α_*S*_ of *Pv*EH1^Y3Z2^ (97.5%) was higher than that of *St*EH1 (97%) and *mb*EH A (79%) [4, 25]. Combining with the research by Kotik et al. [13] we surmised that with the beneficial amino acid exchanging, the position of nucleophilic attack from *Pv*EH1^Y3Z2^ is switched from C_β_ to C_α_ of the oxirane ring of (*S*)-*m*CSO. On the other hand, nucleophilic attack of the oxirane ring of (*R*)-*m*CSO remained largely unaltered in the *Pv*EH1^Y3Z2^, which creates an enantioconvergence towards *rac*-*m*CSO.

### Enantioconvergent hydrolysis of *rac*-*m*CSO by *E. coli*/*pveh1*^Y3Z2^

**Fig. 5:**
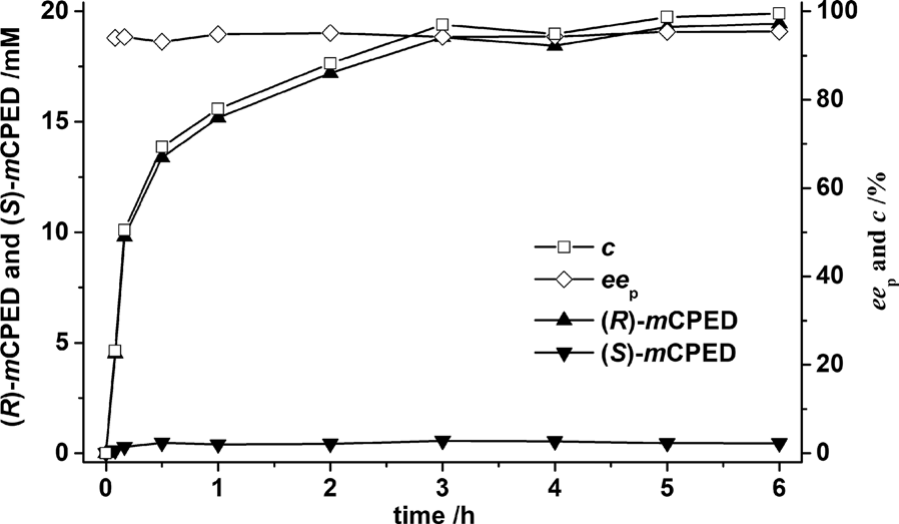
The reaction course of enantioconvergent hydrolysis of *rac*-*m*CSO by whole cells which expressed *Pv*EH1^Y3Z2^. The hydrolysis of *m*CSO was carried out at 25 °C, 2 mL volume containing 20 mM *rac*-*m*CSO, 100 mg/mL *E. coli*/*pv*eh1^Y3Z2^ wet cells until the conversion reached over 99%. The reaction course of aliquots of sample were drawn out periodically, extracted with ethyl acetate, and analyzed by chiral HPLC and GC

To confirm the details in the improvement in enantioconvergence of *Pv*EH1^Y3Z2^, the *ee*_p_, *c*, (*R*)- and (*S*)-*m*CPED concentrations in the enantioconvergent hydrolysis of *rac*-*m*CSO catalyzed by *E. coli*/*pveh1*^Y3Z2^ were monitored by chiral HPLC. With 20 mM *rac*-*m*CSO and 100 mg/mL *E. coli*/*pveh1*^Y3Z2^ adding into a volume of 5.0 mL, hydrolysis of *rac*-*m*CSO was carried out by resting cells at 25 °C, until *c* reached over 99%. With a large amount of (*R*)-*m*CPED produced, few (*S*)-*m*CPED was formed, which decreased *ee*_p_ value. Among the hydrolysis, the *ee*_p_ was all over 93%. The *rac*-*m*CSO was transformed into *m*CPED within 6 h completely (*c* = 99.5%). According to the Fig. 5, at the end of the hydrolysis, the *ee*_p_ and yield were 95.2% and 97.2%, respectively, and the chiral HPLC spectra of production was seen in Additional file 1: Figure S1b. By now, among all the reported EHs which the author has ever known, *Pv*EH1^Y3Z2^ owned the highest enantioconvergence towards *rac*-*m*CSO, even higher than that of *St*EH1 (*ee*_p_ 91%) and Kau2 (*ee*_p_ 92%) [4, 22] (Table 2). Compared with enantioconvergences of other EHs towards other substrates, that of *Pv*EH1^Y3Z2^ also displayed an excellent enantioconvergence. On the other hand, compared with the laboratory evolution of *An*EH from *Aspergillus niger* M200 by Kotik et al. [13] less screening effort was taken and more improvement in enantioconvergence was achieved in this work (*ee*_p_ from 1.0 to 95.2% vs. from 3 to 70.5%).

**Table 2:** Chart of different EHs with high enantioconvergence

EH	Substrate	*ee*_p_/%	Product	References
*Pv*EH1^Y3Z2^	*m*CSO	95.4	(*R*)-*m*CPED	This work
*St*EH1	*m*CSO	91	(*R*)-*m*CPED	[4]
*Vr*EH2^M263N^	*p*NSO	98	(*R*)-*p*NPED	[21]
Kau2	*m*CSO	92	(*R*)-*m*CPED	[22]
Kau2 F:13-B11	*p*CSO	97	(*R*)-*p*CPED	[26]
*Pv*EH3^G170E/F187I^	*p*CSO	92.8	(*R*)-*p*CPED	[27]

## Conclusions

In this work, the enantioconvergence towards *m*CSO was conferred on *Pv*EH1 by a laboratory evolution. Firstly, after identified and analyzed using a CAVER 3.0 software, the SBP of *Pv*EH1 was studied by leucine scanning. The result shows that *Pv*EH1^Y149L^ and *Pv*EH1^P184L^ have a marked increase in enantioconvergence, which means that sites 149 and 184 played important roles in the enantioconvergent hydrolysis of *rac*-*m*CSO. Secondly, to confirm the best residue at each site, saturation mutagenesis libraries *E. coli*/*pveh1*^Y149X^ and *E. coli*/*pveh1*^P184X^ were constructed and screened. Mutants *Pv*EH1^Y149L^ and *Pv*EH1^P184W^ have the highest enantioconvergence in each saturation mutagenesis library. Thirdly, mutant *Pv*EH1^Y3Z2^ containing five residue replacements was constructed using *Pv*EH1^Y3^ as the template. The enantioconvergence of *Pv*EH1^Y3Z2^ was 94-fold higher than that of *Pv*EH1. The analysis of regioselectivity coefficients indicated that the position of nucleophilic attack from mutant *Pv*EH1^Y3Z2^ switched from C_β_ to C_α_ of the oxirane ring of (*S*)-*m*CSO, which led to the improvement in enantioconvergence. At last, enantioconvergent hydrolysis by *E. coli/pveh1*^Y3Z2^ was monitored. The result showed that the *ee*_p_ and yield of (*R*)-*m*CPED were 95.2% and 97.2%, when the *rac*-*m*CSO was transformed into *m*CPED completely (*c* = 99.5%).

## Supplementary information

**Additional file 1: Figure S1.** Chiral HPLC spectra for enantioconvergence hydrolytic of *rac*-*m*CSO by *Pv*EH1^Y3Z2^ including analysis of *rac*-*m*CSO and *rac*-*m*CPED (a), and analysis of (*R*)-*m*CPED (b). **Figure S2.** The comparison of the homology model (red) with the template model (green). **Figure S3.** The change in RMSD values of the whole model. The RMSD value of the whole model tends to 0.18 nm. **Figure S4.** The Ramachandran plots of the model. The Ramachandran favored residue sites was 96.07%, which means that the most distribution of residues is good and the model could be believed. **Figure S5.** The local quality estimate of every residue. Compared with the template model, the identity of amino acids lining the substrate-binding pocket was 93.94%. The local similarity value of most residue in the substrate-binding pocket was over 0.8, which means the residue was highly similar to the template model. **Table S1.** Enantioconvergences and activities of *Pv*EH1 and its three-site mutant towards *rac*-*m*CSO. **Table S2.** PCR primers used for the site-saturation saturation mutagenesis of *pveh1*. **Table S3.** PCR primers used for combinatorial site-directed mutagenesis of *pveh1*^*Y3*^. **Table S4.** Catalytic characteristics of representative mutants from leucine scanning mutagenesis.

## Data Availability

All data generated and/or analysed during this study are included in this article.

## References

[1] Xu LN, Fang GY, Yu YH, Ma YF, Ye ZH, Li ZY (2018). Molecular mechanism of heterogeneous supramolecular catalysis of metal-free cucurbituril solid for epoxide alcoholysis. Mol Catal.

[2] Kamble MP, Yadav GD (2018). Biocatalytic resolution of (R,S)-styrene oxide using a novel epoxide hydrolase from red mung beans. Catal Today.

[3] Tan CL, Zhang X, Zhu ZJ, Xu MJ, Yang TW, Osire T, Yang ST, Rao ZM (2019). Asp305Gly mutation improved the activity and stability of the styrene monooxygenase for efficient epoxide production in *Pseudomonas putida* KT2440. Microb Cell Fact.

[4] Monterde MI, Lombard M, Archelas A, Cronin A, Arand M, Furstoss R (2004). Enzymatic transformations. Part 58: enantioconvergent biohydrolysis of styrene oxide derivatives catalysed by the *Solanum tuberosum* epoxide hydrolase. Tetrahedron Asymmetry.

[5] Kotik M, Archelas A, Wohlgemuth R (2012). Epoxide hydrolases and their application in organic synthesis. Curr Org Chem.

[6] Kamble MP, Yadav GD (2017). Kinetic resolution of (R,S) phenyl glycidyl ether by red mung beans (*Vigna angularis*) epoxide hydrolases. Biocatal Agric Biotechnol.

[7] Solares LF, Mateo C (2013). Improvement of the epoxide hydrolase properties for the enantioselective hydrolysis of epoxides. Curr Org Chem.

[8] Wu YW, Kong XD, Zhu QQ, Fan LQ, Xu JH (2015). Chemoenzymatic enantioconvergent hydrolysis of *p*-nitrostyrene oxide into (*R*)-*p*-nitrophenyl glycol by a newly cloned epoxide hydrolase *Vr*EH2 from *Vigna radiate*. Catal Commun.

[9] Li C, Zhao J, Hu D, Hu BC, Wang R, Zang J, Wu MC (2019). Multiple site-directed mutagenesis of a *Phaseolus vulgaris* epoxide hydrolase to improve its catalytic performance towards *p*-chlorostyrene oxide based on the computer-aided re-design. Int J Biol Macromol.

[10] Ye HH, Hu D, Shi XL, Wu MC, Deng C, Li JF (2016). Directed modification of a novel epoxide hydrolase from *Phaseolus vulgaris* to improve its enantioconvergence towards styrene epoxides. Catal Commun.

[11] Li AT, Qu G, Sun ZT, Reetz MT (2019). Statistical analysis of the benefits of focused saturation mutagenesis in directed evolution based on reduced amino acid alphabets. ACS Catal.

[12] Hibbert EG, Dalby PA (2005). Directed evolution strategies for improved enzymatic performance. Microb Cell Fact.

[13] Kotik M, Archelas A, Faměrová V, Oubrechtová P, Křen V (2011). Laboratory evolution of an epoxide hydrolase—towards an enantioconvergent biocatalyst. J Biotechnol.

[14] Zheng HB, Kahakeaw D, Acevedo JP, Reetz MT (2010). Directed evolution of enantioconvergency: the case of an epoxide hydrolase-catalyzed reaction of a racemic epoxide. ChemCatChem.

[15] Sun ZT, Lonsdale R, Kong XD, Xu JH, Zhou JH, Reetz MT (2015). Reshaping an enzyme binding pocket for enhanced and inverted stereoselectivity: use of smallest amino acid alphabets in directed evolution. Angew Chem Int Ed.

[16] Kong XD, Yuan S, Li L, Chen S, Xu JH, Zhou J (2014). Engineering of an epoxide hydrolase for efficient bioresolution of bulky pharmaco substrates. Proc Natl Acad Sci.

[17] Zou SP, Zheng YG, Wu Q, Wang ZC, Xue YP, Liu ZQ (2018). Enhanced catalytic efficiency and enantioselectivity of epoxide hydrolase from *Agrobacterium radiobacter* AD1 by iterative saturation mutagenesis for (*R*)-epichlorohydrin synthesis. Appl Microbiol Biotechnol.

[18] Kille S, Acevedo-Rocha CG, Parra LP, Zhang ZG, Opperman DJ, Reetz MT, Acevedo JP (2013). Reducing codon redundancy and screening effort of combinatorial protein libraries created by saturation mutagenesis. ACS Synth Biol.

[19] Li C, Hu D, Zong XC, Deng C, Feng L, Wu MC, Li JF (2017). Asymmetric hydrolysis of styrene oxide by *Pv*EH2, a novel *Phaseolus vulgaris* epoxide hydrolase with extremely high enantioselectivity and regioselectivity. Catal Commun.

[20] Barth S, Fischer M, Schmid RD, Pleiss J (2004). Sequence and structure of epoxide hydrolases: a systematic analysis. Proteins.

[21] Li FL, Kong XD, Chen Q, Zheng YC, Xu Q, Chen FF, Fan LQ, Lin GQ, Zhou JH, Yu HL, Xu JH (2018). Regioselectivity engineering of epoxide hydrolase: near-perfect enantioconvergence through a single site mutation. ACS Catal.

[22] Kotik M, Stepánek V, Grulich M, Kyslík P, Archelas A (2010). Access to enantiopure aromatic epoxides and diols using epoxide hydrolases derived from total biofilter DNA. J Mol Catal B Enzym.

[23] Amrein BA, Bauer P, Duarte F, Carlsson ÅJ, Naworyta A, Mowbray SL, Widersten M, Kamerlin SCL (2015). Expanding the catalytic triad in epoxide hydrolases and related enzymes. ACS Catal.

[24] Reetz MT (2013). The importance of additive and non-additive mutational effects in protein engineering. Angew Chem Int Ed.

[25] Xu W, Xu JH, Pan J, Gu Q, Wu XY (2006). Enantioconvergent hydrolysis of styrene epoxides by newly discovered epoxide hydrolases in mung bean. Org Lett.

[26] Kotik M, Zhao W, Iacazio G, Archelas A (2013). Directed evolution of metagenome-derived epoxide hydrolase for improved enantioselectivity and enantioconvergence. J Mol Catal B Enzym.

[27] Hu BC, Li C, Wang R, Zong XC, Li JP, Li JF, Wu MC (2018). Improvement in the activity and enantioconvergency of *Pv*EH3, an epoxide hydrolase from *Phaseolus vulgaris*, for *p*-chlorostyrene oxide by site-saturation mutagenesis. Catal Commun.

