# Supplementary material for: Substantially improving the enantioconvergence of PvEH1, a Phaseolus vulgaris epoxide hydrolase, towards m-chlorostyrene oxide by laboratory evolution
Source: Microb Cell Fact. 2019 Nov 18;18:202. doi: 10.1186/s12934-019-1252-4 (PMC6859628; doi:10.1186/s12934-019-1252-4)
Supplement: Supplementary file 1 — Additional file 1: Figure S1. Chiral HPLC spectra for enantioconvergence hydrolytic of rac-mCSO by PvEH1Y3Z2 including analysis of rac-mCSO and rac-mCPED (a), and analysis of (R)-mCPED (b). Figure S2. The comparison of the homology model (red) with the template model (green). Figure S3. The change in RMSD values of the whole model. The RMSD value of the whole model tends to 0.18 nm. Figure S4. The Ramachandran plots of the model. The Ramachandran favored residue sites was 96.07%, which means that the most distribution of residues is good and the model could be believed. Figure S5. The local quality estimate of every residue. Compared with the template model, the identity of amino acids lining the substrate-binding pocket was 93.94%. The local similarity value of most residue in the substrate-binding pocket was over 0.8, which means the residue was highly similar to the template model. Table S1. Enantioconvergences and activities of PvEH1 and its three-site mutant towards rac-mCSO. Table S2. PCR primers used for the site-saturation saturation mutagenesis of pveh1. Table S3. PCR primers used for combinatorial site-directed mutagenesis of pveh1Y3. Table S4. Catalytic characteristics of representative mutants from leucine scanning mutagenesis. [file 12934_2019_1252_MOESM1_ESM.docx]

**Appendix S1. Additional data**

Substantially improving the enantioconvergence of *Pv*EH1, a *Phaseolus vulgaris* epoxide hydrolase, towards *m*-chlorostyrene oxide by laboratory evolution

Xun-Cheng Zong ^1^, Chuang Li ^1^, Yao-Hui Xu ^2^, Die Hu ^3^, Bo-Chun Hu ^1^, Jia Zang ^2,^*, Min-Chen Wu ^3,^*

^1^ Key Laboratory of Carbohydrate Chemistry and Biotechnology, Ministry of Education, School of Biotechnology, Jiangnan University, Wuxi 214122, China

^2^ The Affiliated Wuxi Matemity and Child Health Care Hospital of Nanjing Medical University, Wuxi 214002, China

^3^ Wuxi School of Medicine, Jiangnan University, Wuxi 214122, China

* Corresponding authors.

*E-mail addresses:* zangj1976@sina.com (J. Zang); biowmc@126.com (M.-C. Wu).


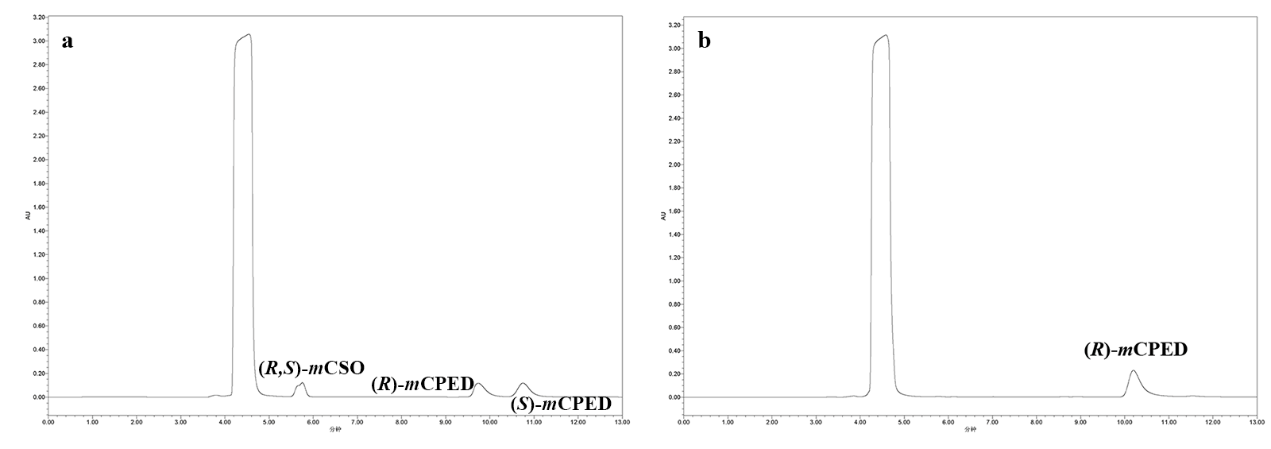


**Figure S1.** Chiral HPLC spectra for enantioconvergence hydrolytic of *rac*-*m*CSO by *Pv*EH1^Y3Z2^ including analysis of *rac*-*m*CSO and *rac*-*m*CPED (a), and analysis of (*R*)-*m*CPED (b).


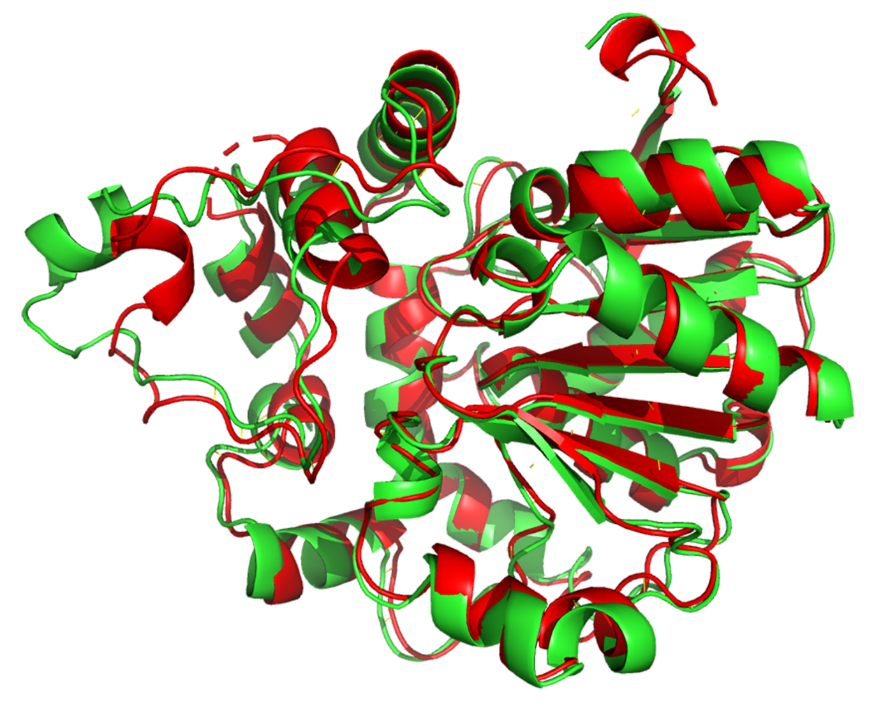


**Figure S2.** The comparison of the homology model (red) with the template model (green).





**Figure S3.** The change in RMSD values of the whole model. The RMSD value of the whole model tends to 0.18 nm.


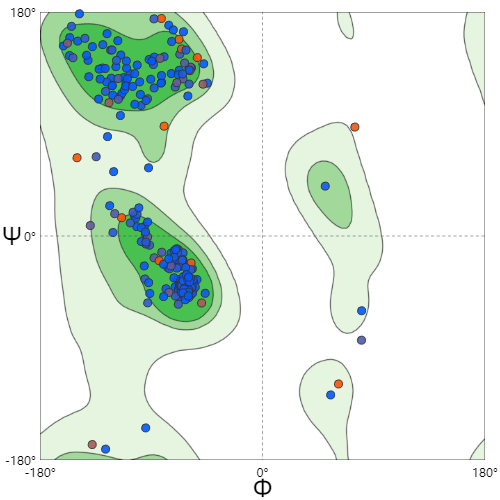


**Figure S4.** The Ramachandran plots of the model. The Ramachandran favored residue sites was 96.07%, which means that the most distribution of residues is good and the model could be believed [1].


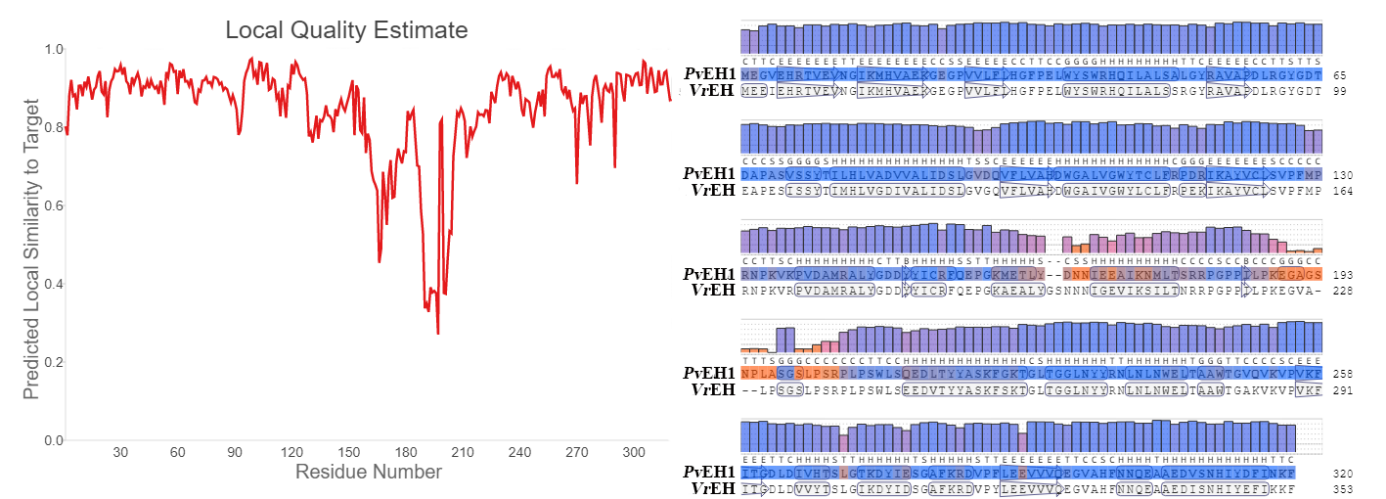


**Figure S5.** The local quality estimate of every residue. Compared with the template model, the identity of amino acids lining the substrate-binding pocket was 93.94%. The local similarity value of most residue in the substrate-binding pocket was over 0.8, which means the residue was highly similar to the template model.

**Table S1**

Enantioconvergences and activities of *Pv*EH1 and its three-site mutant towards *rac*-*m*CSO.

| EH | β*_R_* (%) | α*_S_* (%) | *ee*_p_ (%) | Activity (U/g wet cell) |
| --- | --- | --- | --- | --- |
| *Pv*EH1 | 94.1 | 10.3 | 1.0 | 5.83 ± 0.26 |
| *Pv*EH1^Y3^ | 94.4 | 75.3 | 69.7 | 5.16 ± 0.23 |

**Table S2**

PCR primers used for the site-saturation saturation mutagenesis of *pveh1*.

| Mixed primer^a^ | | Oligonucleotide sequence (5′→3′)^b^ | Size (bp) |
| --- | --- | --- | --- |
| Y149X-F | Y149NDT-F | GGGGATGACNDTTACATTTGCAGATTC | 27 |
|  | Y149VHG-F | GGGGATGACVHGTACATTTGCAGATTC | 27 |
|  | Y149TGG-F | GGGGATGACTGGTACATTTGCAGATTC | 27 |
| P184X-F | P184NDT-F | GAGACCAGGACCANDTATACTCCCCAA | 27 |
|  | P184VHG-F | GAGACCAGGACCAVHGATACTCCCCAA | 27 |
|  | P184TGG-F | GAGACCAGGACCATGGATACTCCCCAA | 27 |
| Y149X-R | Y149AHN-R | GAATCTGCAAATGTAAHNGTCATCCCC | 27 |
|  | Y149CDB-R | GAATCTGCAAATGTACDBGTCATCCCC | 27 |
|  | Y149CCA-R | GAATCTGCAAATGTACCAGTCATCCCC | 27 |
| P184X-R | P184NDT-R | TTGGGGAGTATAHNTGGTCCTGGTCTC | 27 |
|  | P184VHG-R | TTGGGGAGTATCDBTGGTCCTGGTCTC | 27 |
|  | P184TGG-R | TTGGGGAGTATCCATGGTCCTGGTCTC | 27 |

^a^ NDT, VHG and TGG were mixed at the ratio of 12:9:1, while AHN, CDB and CCA at the same ratio.

^b^ The codons encoding mutation residues were boxed. The mixture of NDT (N=A/T/G/C, D=A/G/T), VHG (V=A/C/G, H=A/C/T) and TGG (or AHN, CCA and CDB (B=C/G/T)) contained 22 codons coding for the 20 amino acids.

**Table S3**

PCR primers used for combinatorial site-directed mutagenesis of *pveh1^Y3^*.

| Primer name | Oligonucleotide sequence (5′→3′)^a^ | Size (bp) |
| --- | --- | --- |
| Y149L-F | GGGGATGACCTGTACATTTGCAGATTC | 27 |
| Y149L-R | GAATCTGCAAATGTAGACGTCATCCCC | 27 |
| P184W-F | GAGACCAGGACCATGGATACTCCCCAA | 27 |
| P184W-R | TTGGGGAGTATCCATGGTCCTGGTCTC | 27 |

^a^ The codons encoding mutation residues were boxed.

**Table S4**

Catalytic characteristics of representative mutants from leucine scanning mutagenesis.

| Enzyme | *ee*_p_ (%)^a^ | Activity (U/g wet cell) | *c (*%) |
| --- | --- | --- | --- |
| *Pv*EH1 | 1.0 ± 0.05 | 5.8 ± 0.21 | >99 |
| *Pv*EH1^V126L^ | 3.7 ± 0.11 | 2.8 ± 0.09 | >99 |
| *Pv*EH1^M129L^ | –9.5 ± 0.38 | 9.7 ± 0.29 | >99 |
| *Pv*EH1^P137L^ | ND^b^ | ND | ND |
| *Pv*EH1^M141L^ | –1.9 ± 0.11 | 5.2 ± 0.16 | >99 |
| *Pv*EH1^Y149L^ | 55.1 ± 1.72 | 1.2 ± 0.05 | 94 ± 2.5 |
| *Pv*EH1^M160L^ | 3.5 ± 0.13 | 8.7± 0.26 | >99 |
| *Pv*EH1^T162L^ | –8.5 ± 0.35 | 4.3 ± 0.12 | >99 |
| *Pv*EH1^M175L^ | 6.30 ± 0.19 | 6.1 ± 0.13 | >99 |
| *Pv*EH1^P184L^ | 27.2 ± 0.82 | 6.1 ± 0.13 | >99 |

^a^: –*ee*_p_ means that the main diol product is (*S*)-*m*CPED, while +*ee*_p_ means that the main diol product is (*R*)-*m*CPED.

^b^: ND means not detected.

**References**

[1] Chen VB, Arendall WB, Headd JJ, Keedy DA, Immormino RM, Kapral GJ, L. Murray W, Richardson JS, Richardson DC, MolProbity: all-atom structure validation for macromolecular crystallography. Acta. Crystallogr D. 2010;45:313–319.
